# Risk Factors for Recurrence and In-Hospital Mortality in Patients with *Clostridioides difficile*: A Nationwide Study

**DOI:** 10.3390/jcm14144907

**Published:** 2025-07-10

**Authors:** Rafael Garcia-Carretero, Oscar Vazquez-Gomez, Belen Rodriguez-Maya, Ruth Gil-Prieto, Angel Gil-de-Miguel

**Affiliations:** 1 Department of Internal Medicine, Mostoles University Hospital, 28935 Madrid, Spain; govaz5@hotmail.com (O.V.-G.); belenrmaya@gmail.com (B.R.-M.); 2 Department of Preventive Medicine and Public Health, Rey Juan Carlos University, 28922 Madrid, Spain; ruth.gil@urjc.es (R.G.-P.); angel.gil@urjc.es (A.G.-d.-M.)

**Keywords:** *Clostridioides difficile* infection, CDI incidence, epidemiology, outcomes, mortality, recurrence, machine learning

## Abstract

**Background**: *Clostridioides difficile* infection (CDI) is a major cause of healthcare-associated morbidity and mortality. Understanding the predictors of in-hospital mortality and recurrence of CDI is key for improving outcomes. This study combined traditional statistical methods and machine learning approaches to identify risk factors for these outcomes. **Methods**: We conducted a nationwide, retrospective study using the Spanish Minimum Basic Data Set at Hospitalization, analyzing 34,557 admissions with CDI from 2020 to 2022. Logistic regression combined with the least absolute shrinkage and selection operator (LASSO) was used to identify the most relevant predictors. Survival analyses using Cox regression and LASSO were also performed to assess time-to-mortality predictors. **Results**: Mortality and recurrence rates increased over the study period, reflecting the increasing burden of CDI. LASSO identified a parsimonious subset of predictors while maintaining predictive accuracy (area under the curve: 0.71). Older age (OR = 2.10, 95%CI: 1.98–2.22), Charlson Comorbidity Index ≥ 2 (OR = 1.42, 95%CI: 1.33–1.52), admission to the intensive care unit (OR = 3.09, 95%CI: 2.88–3.32), congestive heart failure (OR = 1.71, 95%CI: 1.61–1.82), malignancies (OR = 1.76, 95%CI: 1.66–1.87), and dementia (OR = 1.36, 95%CI: 1.25–1.48) were strongly associated with all-cause hospital mortality. For recurrence, age ≥ 75 years (OR = 1.19, 95%CI: 1.12–1.27), chronic kidney disease (OR = 1.15, 95%CI: 1.08–1.23), and chronic liver disease (OR = 1.43, 95%CI: 1.16–1.74) were the strongest predictors, while malignancy appeared protective, likely due to survivor bias. **Conclusions**: Our study provides a robust framework for predicting CDI outcomes. The integration of traditional statistical methods and machine learning applied to a large dataset may improve the reliability of the results. Our findings highlight the need for targeted interventions in high-risk populations and emphasize the potential utility of machine learning in risk stratification. Future studies should validate these models in external cohorts and explore survivor bias in malignancy-associated outcomes.

## 1. Introduction

*Clostridioides difficile* infection (CDI) is an important global healthcare challenge associated with significant morbidity, mortality, and economic burden in hospitalized patients [1,2]. *C. difficile* is a pathogen that can cause significant gastrointestinal illness, particularly in individuals with disrupted gut microbiota. As a healthcare-associated pathogen primarily affecting hospitalized patients, it has emerged as a public health concern, having increased in incidence and complexity over the past two decades [3,4].

The epidemiology of CDI is complicated by several key factors, including different patterns of virulence, antibiotic resistance, and the complex interplay between patient characteristics and the host’s immune response, particularly among the elderly [5,6]. Despite advances in hospital healthcare, including newer drugs like fidaxomicin and bezlotoxumab [7,8], CDI remains a significant clinical challenge, especially for vulnerable patients. It is associated with prolonged hospital stays [9], increased healthcare costs [10,11], and substantial patient morbidity [12].

Mortality and recurrence are critical outcomes with significant implications for patient prognosis and healthcare resource utilization [11]. Therefore, a thorough understanding of the features and risk factors that contribute to these adverse outcomes is essential for the development of prevention and management strategies. Established risk factors for mortality include older age, chronic kidney disease, chronic liver disease, and cardiovascular disease [13]. However, complex interactions among patient and pathogen characteristics, as well as environmental factors and the development of antibiotic resistance, make it difficult to fully understand and predict adverse outcomes associated with CDI [14]. Indeed, to date, there is no widely accepted model for predicting CDI mortality or recurrence. Most previous predictive models have shown poor performance due to the use of constrained parametric methods (e.g., linear regression) or by not considering interactions among variables. These shortcomings can lead to models that select a subset of interpretable features but fail to fully explain the outcomes. However, a recently reported model that applied a machine-learning (ML) method utilizing recurrent neural networks [15] reported good predictive results, suggesting that ML methods could improve predictive performance, warranting further investigation into their application to CDI outcomes. Understanding the epidemiology and exploring the underlying comorbidities associated with the poor outcomes can be crucial to tackling the burden of CDI. This study aims to address gaps in the current literature regarding predictive models for mortality and recurrence due to *C. difficile* infections.

Therefore, in this study, we analyzed relevant risk factors associated with in-hospital mortality in CDI patients as well as with CDI recurrence (rCDI). Then we used an ML method to identify the most robust set of such risk factors. Ultimately, we aimed to develop an interpretable, accurate, predictive model that can provide insights into clinical decision-making and risk stratification for patients vulnerable to severe CDI outcomes.

## 2. Materials and Methods

### 2.1. Study Design, Population, and Data Collection

We conducted a nationwide, observational, retrospective study to describe the epidemiological and clinical characteristics of hospital admissions for CDI in Spain. Data were sourced from the Minimum Basic Data Set at Hospitalization (MBDS-H), a mandatory, centralized registry of the Spanish National Health System. This database is a standardized, national registry of discharge reports from nearly 95% of Spanish hospitals (both public and private) that provides valuable demographic and clinical information (including age, sex, admission/discharge dates, hospital type, residence, ICU admission, mortality, diagnoses, procedures, and cost). Although primarily used for economic analysis, the MBDS-H also serves as a valuable source of medical information; in fact, it houses an estimated 97% of all discharge reports in the country.

In this registry, medical conditions, diagnoses, and procedures are coded using the 10th Clinical Revision of the International Classification of Diseases (ICD-10-CM) beginning in 2016. Our dataset included hospitalizations from 2020 to 2022.

### 2.2. Selection of Variables

We collected data on approximately 34,000 hospital admissions associated with CDI, identified using ICD-10-CM codes A04.71 and A04.72 (enterocolitis due to *C. difficile*) in any diagnostic position (whether a primary or secondary diagnosis). Prior to 2020, CDI was coded solely as A04.7. Beginning in 2020, this code was replaced by two mutually exclusive codes: A04.71 (recurrent CDI) and A04.72 (CDI not specified as recurrent). To ensure unequivocal identification of CDI cases (recurrent vs. primary), we focused on the period after this coding change.

As independent variables, we included demographics (age, sex), comorbidities (diabetes mellitus, myocardial infarction, congestive heart failure, hypertension, cerebrovascular disease, chronic kidney disease, chronic liver disease, malignancy, obesity, dementia, and HIV infection), and markers of CDI severity, namely, recurrence and admission to an intensive care unit (ICU). All variables of interest and chronic conditions were identified using the applicable code for ICD-10-CM, as mentioned. We also calculated the Charlson Comorbidity Index (CCI) [16], which classifies comorbidities that influence mortality risk. We used the CCI to assess the overall burden of comorbid conditions (i.e., patients’ medical complexity). As dependent variables, we considered recurrence (encoded as A04.71) and mortality (defined as all-cause in-hospital death).

### 2.3. Ethical Considerations

The data utilized herein were anonymized, and all data are publicly available through the Spanish National Health System upon reasonable request [17]. This study was approved by the Ethical Board of Universidad Rey Juan Carlos (ID number 091120245642024). No identifying information was included in the manuscript. Because the authors used historical data and did not contain personal identifying information, the Ethical Board considered informed consent was not necessary. So, the need for informed consent to participate was waived by our Ethical Board. All procedures involving human participants were conducted in accordance with the ethical standards of the responsible institutional and/or national research committee and with the 1964 Helsinki Declaration and its later amendments or comparable ethical standards.

### 2.4. Statistical Analyses

First, we performed descriptive and correlational analyses. Demographic and clinical characteristics were expressed as medians and interquartile ranges (IQRs) for continuous variables and as frequencies and percentages for categorical variables. Correlational analyses were performed using the chi-square test for categorical variables and the Wilcoxon rank sum test for continuous variables. Poisson regression was also used to assess trends in the data over time. We identified risk factors associated with in-hospital mortality using odds ratios (ORs) and hazard ratios (HRs). For recurrence, only ORs were calculated. Multicollinearity was assessed using variance inflation factors (VIFs). Statistical analyses were conducted using R language version 4.4.2 (R Core Team, Vienna, Austria) on a Debian 12 GNU/Linux workstation. For all tests, the level of statistical significance was set at *p* < 0.05. We used packages such as survival, glmnet, and ggplot2 for statistical modeling and visualization.

### 2.5. Multivariate Logistic Regression and Survival Analyses

We used a full-feature model, including all covariates (not just those significant at *p*< 0.05 in correlational analyses), to fit either a binary logistic regression model or a Cox proportional hazards model. Binary logistic regression models were used to calculate beta-coefficients and ORs. As a standard and straightforward approach in biomedical research with binary outcomes, logistic regression facilitates interpretation of the effects of explanatory variables on the response variable and allows for OR calculation. For survival analyses, we used the Cox proportional hazards model (Cox regression) to examine the relationship between predictors and time to in-hospital mortality using the hazard ratio (HR) as a risk-assessment measure. Mortality was considered the dependent variable, and length of stay was used as the time-to-event variable. Patients with hospital stays exceeding 100 days were excluded to ensure data reliability and focus on clinically relevant timeframes. Recurrence was not assessed through survival analyses because time-to-event information was not available. The proportional hazards assumptions were verified using Schoenfeld residuals.

### 2.6. Machine Learning for Feature Selection

When the full-feature logistic or Cox regression models included numerous explanatory variables, resulting in overly complex models, we employed an ML approach to select a subset of features, thereby reducing the number of relevant risk factors and creating a more parsimonious model without sacrificing predictive accuracy or reliability. The full-feature logistic regression model served as the reference (gold standard).

Specifically, we used L1-penalized logistic regression, also known as the least absolute shrinkage and selection operator (LASSO), to select this subset of features, which were subsequently fitted using either a logistic regression or Cox model [18,19], as appropriate. Then ORs or HRs, with 95% confidence intervals (CIs), were calculated. LASSO is recommended by the Transparent Reporting of a Multivariable Prediction Model for Individual Prognosis or Diagnosis (TRIPOD) checklist for developing and validating risk and diagnostic models [20].

Thus, we developed two models: a full-feature logistic regression model and a LASSO-derived model. Model performance was compared using the area under the curve (AUC), with pairwise comparisons based on the DeLong method [21], to identify the more parsimonious model without loss of predictive accuracy.

For survival analysis, we similarly combined LASSO with Cox regression to create a parsimonious model, alongside the full-feature Cox regression model. Model fitting and performance were assessed and compared using the concordance index (C-index) and the Akaike Information Criterion (AIC).

## 3. Results

### 3.1. Descriptive Analyses

**Figure 1:**
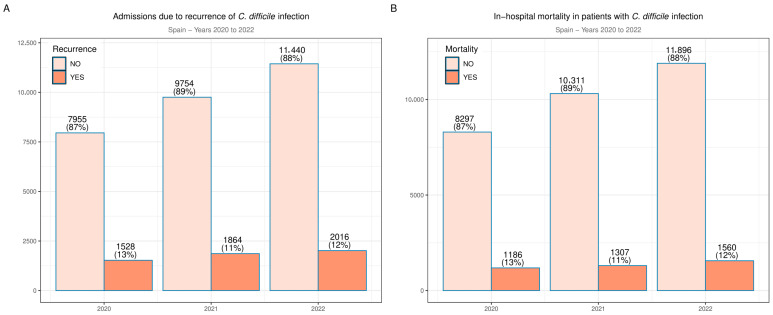
Recurrence rate of CDI (**A**) and evolution of the in-hospital mortality rate over the years (**B**).

We collected data from 34,557 admissions between 2020 and 2022 with a diagnosis of enterocolitis due to *C. difficile*. Table 1 summarizes the results of the descriptive analyses. The median age was 76 years (IQR 62–85). During the 3-year period studied, we observed an increasing number of hospitalizations, with 9483 hospital admissions in 2020 and 13,456 in 2022. This increase was steady in both sexes. Hospitalized women were more frequent among patients presenting with CDI (53% vs. 47%, *p* < 0.001). The percentage of ICU admissions was 9.3%. The median hospital length of stay was 13 days (IQR 7–25). The rate of patients admitted with rCDI was 16% over the entire study period, but there was an increasing trend in the absolute number of patients: 1528 in 2020 vs. 2016 in 2022. A similar phenomenon was observed with deaths: the mortality rate was constant at 12% but absolute numbers increased each year, as shown in Figure 1. Comorbidities were assessed individually, with the most prevalent conditions being hypertension (31%), chronic kidney disease (26%), type 2 diabetes (25%), malignancies (22%), and congestive heart failure (19%). The median CCI was 2 (IQR 1–5).

### 3.2. Mortality

Table 2 shows the results of correlational analyses performed using mortality as the dependent variable. Age was considered both a continuous and categorical variable and was associated with mortality under both treatments (as a continuous variable: median 81, IQR: 71–88, *p* < 0.001; as a categorical variable: age ≥ 75). Being male was also associated with mortality. Mortality was positively correlated with ICU admission, and ICU and hospital length of stay, but negatively correlated with recurrence (16 vs. 13%, *p* < 0.001). All comorbidities except for obesity, chronic liver disease, and HIV infection were positively correlated with mortality. Regarding CCI, it was also considered both as a continuous and as a categorical variable, and in both cases, it was positively correlated with mortality.

### 3.3. Multivariate Analyses Combining Logistic Regression and Machine Learning Algorithms

**Figure 2:**
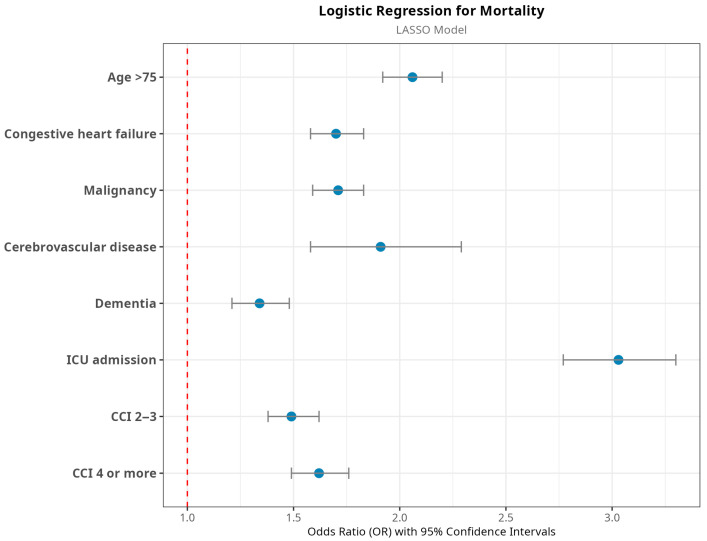
Results of the logistic regression model using the subset of selected variables with the LASSO model. CCI: Charlson Comorbidity Index. Vertical dashed line denotes the OR = 1. Horizontal lines denote odds ratio and confidence intervals.

First, we fitted the standard logistic regression model using all variables (full-feature model), and then we derived variable subsets using the ML algorithm (LASSO). Table 3 presents the results for both models. The full-feature model, after nonsignificant variables were removed, included 10 variables, while the LASSO model selected 7. The AUC was 0.71 for both models. DeLong’s method showed no significant difference in predictive performance between the two. Therefore, given their equivalent predictive performance, we selected the more parsimonious LASSO model, which identified age ≥ 75, congestive heart failure, cerebrovascular disease, malignancies, dementia, CCI ≥ 2, and ICU admission as risk factors. ORs for the LASSO model are visualized in Figure 2.

Although age and CCI, when treated as continuous variables, were significant risk factors for mortality in all models, the results were not straightforward to interpret. Thus, we opted to categorize them to improve interpretability. Age was categorized as <75 or ≥75 (based on the median age), and CCI was divided into three score categories.

### 3.4. Survival Analyses with Cox Regression and Machine Learning Algorithms

**Figure 3:**
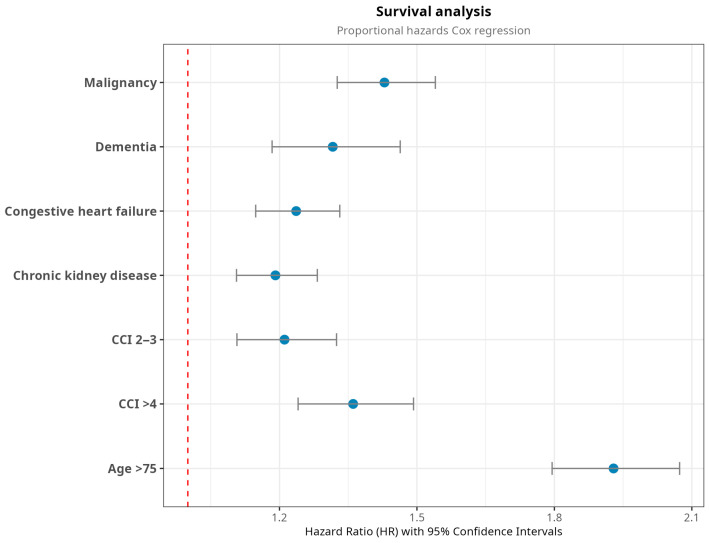
Results of survival analysis performed with the Cox proportional hazard model combined with the LASSO model. CCI: Charlson Comorbidity Index.

Next, we fitted the Cox proportional hazards model to our dataset. As mentioned in the Section 2, we developed two Cox models, using both a standard, full-feature approach and the LASSO algorithm. Using the C-index and AIC as metrics, we selected the LASSO model because it resulted in the most parsimonious model without losing accuracy or performance. LASSO generated a subset that fit well with the data and, combined with the Cox regression, selected congestive heart failure, malignancies, dementia, chronic kidney disease, CCI ≥ 2, and age ≥ 75 as the most relevant risk factors in a time-to-event analysis, as shown in Table 4 and Figure 3.

### 3.5. Recurrence

Table 5 shows the results for patients with rCDI compared to cases admitted with their first episode of CDI. Older age, specifically patients ≥75, was associated with rCDI. ICU admission was negatively correlated with recurrence. Regarding comorbidities, congestive heart failure, cerebrovascular disease, chronic kidney disease, chronic liver disease, malignancies, and CCI were positively correlated with recurrence.

**Figure 4:**
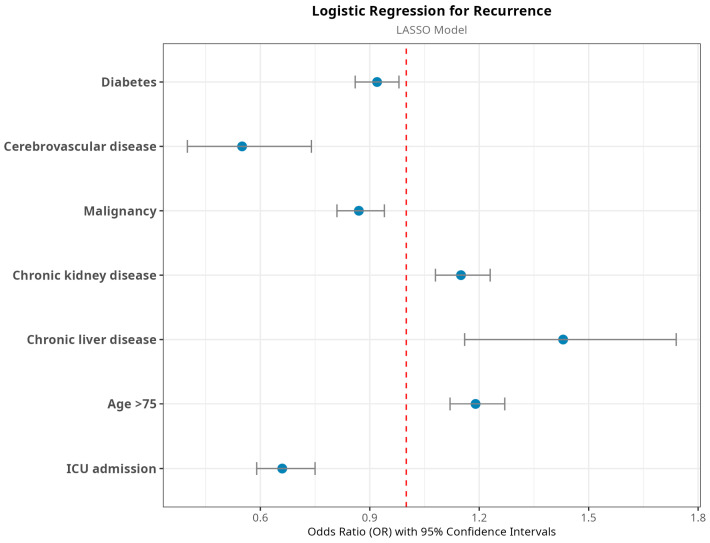
Results of survival analysis performed with the Cox proportional hazard model combined with the LASSO model.

Table 6 shows the results of multivariate analyses predicting recurrence. As in the case of mortality, we used a two-step approach to obtain a parsimonious logistic regression model. First, we compared models that showed similar performance in terms of AUC and accuracy. The full model and the LASSO model had the same performance. We selected the one produced with the LASSO algorithm (it will be discussed later on). It identified age ≥ 75 (OR = 1.19, 95%CI 1.12–1.27, *p* > 0.001), chronic kidney disease (OR = 1.15, 95%CI 1.08–1.23, *p* < 0.001), and chronic liver disease (OR = 1.43, 95%CI 1.16–1.74, *p* > 0.001) as the most relevant predictors. Of note, the model selected the presence of malignancies, diabetes, cerebrovascular disease, and ICU admission as protective predictors. Figure 4 shows a plot of the results of the LASSO model for better visualization.

## 4. Discussion

The aim of our study was to highlight the most important risk factors associated with in-hospital mortality and recurrence in patients with CDI. We used a large, nationwide dataset for robust results. We found that, over the observed 3-year period, CDI-related hospitalizations increased steadily, either as a primary or a recurrent disease. Although both deaths and episodes of rCDI increased in absolute numbers, their rates remained steady (and relatively high in a clinical context). This trend may reflect evolving pathogen virulence, changes in healthcare practices (such as the widespread use of antibiotics), or demographic patterns (such as an aging population). The implications for healthcare systems are key in terms of the associated economic burden [5,11]. Our study also highlights the novel contribution of using ML to refine variable selection in assessments of CDI. By developing and comparing predictive models for recurrence and mortality using different approaches, we not only identified the most relevant risk factors associated with each outcome but were also able to derive more insight from our dataset.

### 4.1. Mortality

Among the two candidate predictive models (full-feature logistic regression and LASSO), the LASSO model demonstrated the best balance of predictive performance (AUC = 0.71) and parsimony. The model indicated that mortality was higher in the elderly and in association with certain comorbidities. Age ≥ 75 was consistently the strongest predictor, associated with approximately double the odds of mortality (OR = 2.10, 95%CI: 1.98–2.22). Other relevant risk factors were congestive heart failure, cerebrovascular disease, malignancies, dementia, ICU admission, and a higher CCI score (≥2). In survival analyses, the parsimonious model achieved with LASSO and combined with Cox regression also outperformed the standard model, identifying the most significant predictors for in-hospital mortality in time-to-event analysis (age ≥ 75, congestive heart failure, malignancies, dementia, chronic kidney disease, and CCI ≥ 2).

Our findings indicate that age in combination with certain comorbidities contributes to CDI severity, consistent with previous publications [22,23]. Na et al. [23], in a cohort of 263 participants, identified age and chronic kidney disease as risk factors for severe CDI, defining severity as death due to CDI, CDI as a contributing factor in mortality, ICU admission, toxic megacolon, or colectomy due to CDI. Similarly, Hensgens et al., in a multicenter cohort study, found that older age and higher comorbidity burden (including malignancies, cardiovascular disease, and CCI) were associated with increased mortality risk [22,24], further emphasizing the importance of age and comorbidities in predicting adverse CDI outcomes.

The association between congestive heart failure, malignancies, and dementia, and increased mortality in our study also aligns with the literature. Several studies have highlighted cardiovascular diseases, chronic kidney failure, and malignancies as common risk factors for all-cause mortality [25,26,27]. Enoch et al. [28] reported similar findings of higher mortality rates associated with dementia, renal disease, and malignancies in patients hospitalized with CDI. Our identification of ICU admission as a significant predictor of all-cause mortality is also in line with the Society for Healthcare Epidemiology of America/Infectious Diseases Society of America severity criteria, which recognize ICU admission as a strong indicator of poor CDI outcomes [14]. These conditions likely contribute to poorer outcomes due to patients’ immune status and frailty, or by exacerbating the physiological stress of CDI [23].

### 4.2. Recurrence

We found that age ≥ 75 and chronic kidney disease were the most relevant risk factors for rCDI. Diabetes, chronic liver disease, and cerebrovascular disease were also selected as important in both models (logistic regression and LASSO). Our findings align with other studies that have reported that older age and chronic kidney disease are associated with higher recurrence rates, likely due to age-related immunosuppression and the impact of renal disease on antibiotic metabolism and gut microbiota [26,27,29].

However, in contrast to expectations, we found that malignancies were a protective factor against rCDI across all models (e.g., OR = 0.87, 95%CI: 0.81, 0.94). Malignancies are commonly associated with immunosuppression and increased risk of infection recurrence due to chemotherapy, immune dysregulation, or prolonged antibiotic exposure [26]. However, in our dataset, malignancies consistently showed an inverse relationship with rCDI. This was also reported by Enoch et al. [28], who hypothesized that patients with malignancies who died during their first CDI episode would not be captured in recurrence data, potentially skewing the results. Although we have no clear explanation, we can hypothesize that our finding may reflect survivor bias; that is, patients with malignancies who develop rCDI represent a subset of individuals with inherently better baseline health. Hence, the most severe patients may have passed away due to the primary episode of CDI, enabling the healthier subset to survive long enough to experience and potentially recover from recurrence. If this were the case, patients with malignancies who died during their first.

A CDI episode would not have been captured in the recurrence dataset, disproportionately representing those with more favorable outcomes. It is also possible that our study design and dataset characteristics may have influenced this result, as also pointed out by Enoch et al. [28]. Also, a Swedish population-based study including over 43,000 individuals studied the role of different types of malignancy in rCDI [30]. It found lower odds of rCDI among patients with ongoing malignancy. This study suggests malignancy is a serious complication for mortality in CDI, but not for rCDI. The authors suggested that many patients may die before recurrence can occur, artificially lowering the observed recurrence rate. So, mortality could mask mortality rates. As in this Swedish study, we acknowledge that our study lacked data on chemotherapy or antibiotics (i.e., unmeasured confounders). Since recurrence could not be adjusted to some variables, our analyses could not accurately capture the association with malignancy, potentially diluting it. We are aware that we relied on an administrative registry and performed retrospective research, which may have amplified this survivor bias.

We also observed a negative correlation between ICU admission and rCDI. While ICU admission was associated with higher mortality, our results suggest that it can reduce the likelihood of recurrence. This could be due to the fact that patients requiring ICU care may receive more aggressive treatment or closer monitoring, thus reducing the risk of recurrence. Another hypothesis is that it may reflect the higher mortality rate in this group, again showing survivor bias, as fewer survivors experience recurrence. This negative correlation contrasts with a previous study that found that ICU admission was associated with both higher mortality and higher recurrence rates [6]. This may suggest that the relationship between ICU care and recurrence may be complex and context-dependent, and deserves further investigation.

Currently, predicting recurrence is a challenging task. Identifying and establishing risk factors for recurrent infection has marginal value in developing reliable models. A recent publication by Boone et al. demonstrated that current clinical scores performed very poorly in predicting recurrence [31]. Likely, existing clinical models, including those based on machine learning, are incapable of accurately predicting rCDI. The models evaluated and externally validated by Boone et al. showed AUROCs very close to that of a random chance. This, in turn, suggests that the predictive models were overfitted to the initial dataset. These authors proposed that predicting recurrence was very difficult, if not impossible, using only clinical variables.

### 4.3. The Role of Inflammatory Bowel Disease

The relationship between inflammatory bowel disease (IBD) and CDI remains complex, as evidenced by the findings of this study. While existing literature, such as the meta-analysis performed by Balram et al. [32] and the narrative review by Bai et al. [33], identifies IBD as a risk factor for CDI, our results are contradictory when considering mortality and recurrence. In bivariate analyses, we observed a positive correlation between IBD and mortality in CDI patients, suggesting that IBD may contribute to poorer outcomes in this population. However, this association did not persist in multivariate analyses, including logistic regression and Cox regression models. This discrepancy may indicate that the risk of adverse outcomes may be more closely linked to underlying comorbidity rather than IBD itself. Also, our findings suggest the importance of adjusting for potential confounders when evaluating the impact of IBD on CDI outcomes.

Likewise, regarding CDI recurrence, our analysis revealed no significant correlation with IBD in either bivariate or logistic regression analyses. The absence of an association in our study may reflect differences in patient populations, treatment strategies, or follow-up durations. Alternatively, it may suggest that IBD does not independently drive CDI recurrence but instead interacts with other conditions, such as antibiotic use or immune suppression, which were not fully accounted for in our models. Further research is needed to address the mechanisms underlying CDI recurrence in IBD patients and to identify modifiable risk factors.

### 4.4. Strength of Machine Learning Modeling

Our study enhances understanding of CDI outcomes by combining standard statistical techniques with ML approaches, specifically LASSO, to reduce the number of predictors and identify the most relevant variables for the final model. Feature selection, model parsimony, and predictive accuracy were key criteria. Compared to standard logistic and Cox regression, the LASSO models offered the best balance of predictive performance and parsimony, highlighting their utility in clinical research [20]. This approach resulted in a more interpretable model and avoided overfitting in our high-dimensional dataset.

Other studies have also used ML or deep learning for similar aims [2,15]. However, we must address the potential discrepancy between mathematical models and clinical significance for both mortality and recurrence, as relevant features may be excluded. ML algorithms may prioritize nonlinear interactions and feature importance based on splits rather than statistical significance. Consequently, they may exclude variables with less impact on predictions of mortality or recurrence in our dataset, even if they are clinically meaningful. We acknowledge that a purely mathematical approach to model selection can be risky when the chosen model excludes predictors that, while less statistically significant, may have clinical relevance or biological plausibility. This does not imply that these features are not clinically relevant, but rather highlights the importance of clinician involvement in model interpretation. Our ML model is a tool to enhance decision-making, not to replace clinical judgment. For example, while LASSO excluded sex and recurrence as relevant predictors of mortality, this does not negate their potential relevance, as demonstrated by other studies [13,32]. Therefore, we presented a comparison of both models in tables. Our findings underscore the need to interpret model outputs in conjunction with established clinical knowledge.

It is worth noting the contribution of Madden et al. using deep learning and trying to produce an explainable machine learning model as a proof of concept [15]. Their predictive model achieved an AUROC of 0.678, which, although far from ideal for clinical decision-making, outperformed previously published clinical models. The authors acknowledge the lack of additional clinical variables as a limitation of their model.

### 4.5. Strengths and Limitations

One strength of our study is the use of a large, nationwide dataset, which makes our findings more robust. Another strength is the novel application of ML to identify risk factors with relevant predictive value. Our findings align with the literature on CDI all-cause mortality and recurrence, particularly regarding the roles of older age, comorbidities, and ICU admission. However, several limitations must be acknowledged. The main one is due to the nature of our dataset. Since the dataset is based on ICD-10-CM, in regard to CDI, it does not distinguish between community-acquired and hospital-acquired cases. Also, the use of an administrative database to identify CDI episodes is dependent on the quality of discharge report data. Reliance on ICD-10-CM coding may introduce misclassification bias due to variation in coding practices across institutions. Furthermore, while valuable for hospital management, this database lacks potentially important information, such as resource utilization during hospitalization (e.g., diagnostic tests, antibiotic treatments) and unrecorded clinical characteristics. Coding practices or underreporting may introduce bias. Additionally, the retrospective and observational design precludes causal inferences. The absence of individual patient-level data, pathogen-specific factors, and treatment regimens (unavailable in our original dataset) may further limit the reliability of our findings.

### 4.6. Future Research

The protective effect of malignancies against recurrence and the negative correlation between ICU admission and recurrence are novel insights that deserve further investigation. Also, future research should investigate the long-term outcomes of CDI beyond in-hospital mortality, e.g., the 30-day mortality. Future studies should focus on validating the predictive models using external datasets. Prospective, longitudinal studies incorporating microbiological and immunological data could provide a more comprehensive understanding of CDI pathophysiology and can be useful for examining long-term outcomes and interventions.

## 5. Conclusions

This work identifies key demographic and clinical predictors of in-hospital mortality and recurrence in patients with CDI, highlighting the critical role of age, comorbidities, and severity markers. The integration of ML techniques, specifically LASSO regression, proved valuable in improving model parsimony without compromising predictive performance. These findings have important implications for risk stratification and clinical decision-making. This should help clinicians develop more effective prevention and management strategies for vulnerable populations. Our descriptions of these risk factors contribute to the body of knowledge on CDI.

As the burden of CDI continues to increase, healthcare systems must prioritize strategies to reduce recurrence and mortality, including optimizing care for high-risk patients. Further research is needed to assess the complex interplay among patients, pathogens, and environmental factors influencing CDI outcomes.

## Figures and Tables

**Table 1:** Characteristics of the studied cohort of patients with CDI between 2020 and 2022 in Spain.

Characteristic	Total, N = 34,557 ^1^	2020, N = 9483 ^1^	2021, N = 11,618 ^1^	2022, N = 13,456 ^1^	*p*-Value ^2^
Age (years, continuous)	76 (62, 85)	75 (62, 84)	76 (62, 85)	76 (63, 86)	<0.001
Age (years, categorical)					<0.001
<75	16,291 (47%)	4627 (49%)	5441 (47%)	6223 (46%)	
≥75	18,266 (53%)	4856 (51%)	6177 (53%)	7233 (54%)	
Sex (women)	18,229 (53%)	4960 (52%)	6129 (53%)	7140 (53%)	0.53
ICU admissions	3223 (9.3%)	854 (9.0%)	1075 (9.3%)	1294 (9.6%)	0.28
ICU LOS	7 (3, 21)	7 (2, 19)	9 (3, 29)	6 (2, 17)	<0.001
Hospitalization LOS	13 (7, 25)	13 (7, 25)	13 (7, 25)	13 (7, 23)	<0.001
rCDI	5408 (16%)	1528 (16%)	1864 (16%)	2016 (15%)	0.024
Deaths	4053 (12%)	1186 (13%)	1307 (11%)	1560 (12%)	0.015
Comorbidities					
Type 2 diabetes	8702 (25%)	2395 (25%)	2929 (25%)	3378 (25%)	0.96
Obesity	2912 (8.4%)	750 (7.9%)	1022 (8.8%)	1140 (8.5%)	0.067
Coronary disease	3702 (11%)	975 (10%)	1289 (11%)	1438 (11%)	0.16
Heart failure	6466 (19%)	1669 (18%)	2216 (19%)	2581 (19%)	0.005
Hypertension	10,554 (31%)	2985 (31%)	3501 (30%)	4068 (30%)	0.066
Cerebrovascular disease	483 (1.4%)	140 (1.5%)	155 (1.3%)	188 (1.4%)	0.68
Dementia	2579 (7.5%)	716 (7.6%)	893 (7.7%)	970 (7.2%)	0.33
Chronic kidney disease	9119 (26%)	2386 (25%)	3079 (27%)	3654 (27%)	0.003
Chronic liver disease	607 (1.8%)	200 (2.1%)	203 (1.7%)	204 (1.5%)	0.003
Chronic pulmonary disease	3513 (10%)	940 (9.9%)	1177 (10%)	1396 (10%)	0.52
AIDS/HIV infection	253 (0.7%)	86 (0.9%)	70 (0.6%)	97 (0.7%)	0.035
Malignancies	7557 (22%)	2135 (23%)	2524 (22%)	2898 (22%)	0.19
Inflammatory bowel disease	1385 (4.0%)	397 (4.2%)	473 (4.1%)	515 (3.8%)	0.36
Charlson Comorbidity Index					0.46
0–1	12,710 (37%)	3511 (37%)	4313 (37%)	4886 (36%)	
2–3	10,234 (30%)	2820 (30%)	3379 (29%)	4035 (30%)	
≥4	11,613 (34%)	3152 (33%)	3926 (34%)	4535 (34%)	
Median score (IQR)	2.00 (1.00, 5.00)	2.00 (1.00, 5.00)	2.00 (1.00, 5.00)	2.00 (1.00, 5.00)	0.49

^1^ Median (IQR) or frequency (%). IQR: interquartile range. ^2^ Poisson regression. ICU: intensive care unit. LOS: length of stay. rCDI: recurrent C. difficile infection. AIDS: acquired immune deficiency syndrome. HIV: human immunodeficiency virus.

**Table 2:** Bivariate analyses for mortality due to CDI between 2020 and 2022 in Spain.

Mortality			
Characteristic	NO, N = 30,504 ^1^	YES, N = 4053 ^1^	*p*-Value ^2^
Age	75 (61, 85)	81 (71, 88)	<0.001
Age (categorized)			<0.001
<75	14,939 (49%)	1352 (33%)	
≥75	15,565 (51%)	2701 (67%)	
Sex (women)	16,238 (53%)	1991 (49%)	<0.001
ICU admissions	2569 (8.4%)	654 (16%)	<0.001
ICU LOS	7 (3, 20)	10 (3, 24)	0.012
Hospitalization LOS	13 (7, 23)	17 (9, 32)	<0.001
rCDI	4872 (16%)	536 (13%)	<0.001
Comorbidities			
Type 2 diabetes	7545 (25%)	1157 (29%)	<0.001
Obesity	2591 (8.5%)	321 (7.9%)	0.22
Coronary disease	3157 (10%)	545 (13%)	<0.001
Heart failure	5190 (17%)	1276 (31%)	<0.001
Hypertension	9453 (31%)	1101 (27%)	<0.001
Cerebrovascular disease	374 (1.2%)	109 (2.7%)	<0.001
Dementia	2167 (7.1%)	412 (10%)	<0.001
Chronic kidney disease	7772 (25%)	1347 (33%)	<0.001
Chronic liver disease	523 (1.7%)	84 (2.1%)	0.10
Chronic pulmonary disease	3001 (9.8%)	512 (13%)	<0.001
AIDS/HIV infection	231 (0.8%)	22 (0.5%)	0.13
Malignancies	6425 (21%)	1132 (28%)	<0.001
Inflammatory bowel disease	1304 (4.3%)	81 (2.0%)	<0.001
Charlson Comorbidity Index	2.00 (1.00, 4.00)	3.00 (2.00, 6.00)	<0.001
Charlson Comorbidity Index (categorized)			<0.001
0–1	11,774 (39%)	936 (23%)	
2–3	8929 (29%)	1305 (32%)	
≥4	9801 (32%)	1812 (45%)	

^1^ Median (IQR) or frequency (%). ^2^ Pearson’s chi-square test; Wilcoxon rank sum test. ICU: intensive care unit. LOS: length of hospital stay. rCDI: recurrent C. difficile infection. HIV: human immunodeficiency virus.

**Table 3:** Multivariate analyses for mortality using a standard approach (full-feature logistic regression model) and the machine learning method (LASSO).

	Full-Feature LR Model		LASSO Model	
	OR1 (95% CI) ^1^	*p*-Value	OR (95% CI) ^1^	*p*-Value
Age (categorized)		<0.001		<0.001
<75	ref			
≥75	2.06 (1.90, 2.23)		2.10 (1.98, 2.22)	
Sex (men)	1.18 (1.10, 1.27)	<0.001		
Diabetes	0.95 (0.87, 1.03)	0.2		
Myocardial infarction	0.98 (0.88, 1.08)	0.7		
Congestive heart failure	1.76 (1.61, 1.91)	<0.001	1.71 (1.61, 1.82)	<0.001
Hypertension	0.86 (0.79, 0.93)	<0.001		
Cerebrovascular disease	1.87 (1.49, 2.33)	<0.001	1.90 (1.62, 2.22)	<0.001
Obesity	0.93 (0.82, 1.05)	0.3		
Malignancy, including lymphoma and leukemia	1.73 (1.59, 1.88)	<0.001	1.76 (1.66, 1.87)	<0.001
Dementia	1.39 (1.23, 1.56)	<0.001	1.36 (1.25, 1.48)	<0.001
Renal disease	0.99 (0.91, 1.08)	0.9		
Moderate or severe liver disease	1.22 (0.95, 1.54)	0.12		
Chronic pulmonary disease	1.01 (0.91, 1.13)	0.8		
AIDS/HIV	1.28 (0.79, 1.97)	0.3		
Inflammatory bowel disease	0.92 (0.70, 1.19)	0.5		
Recurrence	0.77 (0.70, 0.85)	<0.001		
CCI (categorized)				
0–1	ref		ref	
2–3	1.30 (1.18, 1.43)	<0.001	1.42 (1.33, 1.52)	<0.001
≥4	1.42 (1.27, 1.58)	<0.001	1.59 (1.49, 1.71)	<0.001
ICU admission	2.81 (2.54, 3.11)	<0.001	3.09 (2.88, 3.32)	<0.001
	AUC = 0.71, Accuracy = 0.84		AUC = 0.71, Accuracy = 0.84	

^1^ OR: odds ratio, CI: confidence interval, CCI: Charlson Comorbidity Index, LR: logistic regression, LASSO: least absolute shrinkage and selection operator, AUC: area under the curve, ref: reference.

**Table 4:** Multivariate analysis of in-hospital mortality (as the dependent variable) performed using four Cox proportional hazards models.

	Full-Feature Model		LASSO Model	
	HR (95% CI)	p-Value	HR (95% CI)	p-Value
Sex (men)	1.01 (0.95, 1.08)	0.7		
Diabetes	0.98 (0.90, 1.05)	0.5		
Myocardial infarction	1.08 (0.99, 1.19)	0.10		
Congestive heart failure	1.24 (1.15, 1.34)	<0.001	1.24 (1.15, 1.33)	<0.001
Hypertension	1.02 (0.94, 1.10)	0.7		
Cerebrovascular disease	1.00 (0.82, 1.22)	>0.9		
Obesity	0.93 (0.83, 1.05)	0.2		
Malignancies	1.45 (1.34, 1.56)	<0.001	1.43 (1.33, 1.54)	<0.001
Dementia	1.34 (1.21, 1.49)	<0.001	1.32 (1.18, 1.46)	<0.001
Chronic kidney disease	1.21 (1.12, 1.31)	<0.001	1.19 (1.11, 1.28)	<0.001
Chronic liver disease	1.43 (1.15, 1.77)	0.001		
Chronic pulmonary disease	1.14 (1.03, 1.26)	0.010		
AIDS/HIV	1.03 (0.68, 1.58)	0.9		
Inflammatory bowel disease	0.85 (0.68, 1.06)	0.2		
ICU admission	1.09 (0.99, 1.20)	0.069		
CCI				
0–1	ref			
2–3	1.19 (1.09, 1.31)	<0.001	1.21 (1.11, 1.32)	<0.001
≥4	1.31 (1.18, 1.45)	<0.001	1.36 (1.24, 1.49)	<0.001
Age (categorized)				
<75	ref			
≥75	1.96 (1.82, 2.12)	<0.001	1.93 (1.80, 2.07)	<0.001

HR: hazard ratio, CI: confidence interval, CCI: Charlson Comorbidity Index, ref: reference.

**Table 5:** Bivariate analyses between the studied variables and the recurrence of CDI.

Recurrence			
Characteristic	NO, N = 29,149 ^1^	YES, N = 5408 ^1^	*p*-Value ^2^
Age	75 (62, 85)	78 (66, 86)	<0.001
Age (categorized)			<0.001
<75	14,025 (48%)	2266 (42%)	
≥75	15,124 (52%)	3142 (58%)	
Sex (women)	15,287 (52%)	2942 (54%)	0.008
ICU admissions	2882 (9.9%)	341 (6.3%)	<0.001
ICU LOS	7 (3, 21)	6 (2, 17)	0.040
Hospitalization LOS	13 (7, 25)	11 (6, 22)	<0.001
Comorbidities			
Type 2 diabetes	7352 (25%)	1350 (25%)	0.69
Obesity	2456 (8.4%)	456 (8.4%)	0.99
Coronary disease	3083 (11%)	619 (11%)	0.058
Heart failure	5360 (18%)	1106 (20%)	<0.001
Hypertension	8967 (31%)	1587 (29%)	0.038
Cerebrovascular disease	439 (1.5%)	44 (0.8%)	<0.001
Dementia	2142 (7.3%)	437 (8.1%)	0.060
Chronic kidney disease	7506 (26%)	1613 (30%)	<0.001
Chronic liver disease	484 (1.7%)	123 (2.3%)	0.002
Chronic pulmonary disease	2932 (10%)	581 (11%)	0.13
AIDS/HIV infection	219 (0.8%)	34 (0.6%)	0.33
Malignancies	6515 (22%)	1042 (19%)	<0.001
Inflammatory bowel disease	1159 (4.0%)	226 (4.2%)	0.48
Charlson Comorbidity Index	2 (1, 5)	2 (1, 5)	0.009
CCI (categorized)			0.034
0–1	10,800 (37%)	1910 (35%)	
2–3	8620 (30%)	1614 (30%)	
≥4	9729 (33%)	1884 (35%)	

^1^ Median (IQR) or frequency (%). ^2^ Pearson’s chi-square test; Wilcoxon rank sum test. ICU: intensive care unit. LOS: length of hospital stay. HIV: human immunodeficiency virus.

**Table 6:** Multivariate analyses for recurrence using a standard approach (full-feature logistic regression model) and the machine learning method (LASSO).

	Full-Feature Logistic Regression Model		LASSO Model	
	OR (95% CI) ^1^	*p*-Value	OR (95% CI)	*p*-Value
Age (categorized)				
<75	ref		ref	
≥75	1.18 (1.10, 1.26)	<0.001	1.19 (1.12, 1.27)	<0.001
Sex (men)	0.95 (0.89, 1.01)	0.10		
Diabetes	0.90 (0.84, 0.97)	0.008	0.92 (0.86, 0.98)	0.013
Myocardial infarction	1.04 (0.95, 1.15)	0.4		
Congestive heart failure	1.00 (0.92, 1.09)	>0.9		
Hypertension	0.95 (0.89, 1.02)	0.2		
Cerebrovascular disease	0.54 (0.39, 0.73)	<0.001	0.55 (0.40, 0.74)	<0.001
Obesity	0.99 (0.89, 1.10)	0.8		
Malignancy, including lymphoma and leukemia	0.86 (0.79, 0.93)	<0.001	0.87 (0.81, 0.94)	<0.001
Dementia	0.99 (0.88, 1.10)	0.8		
Renal disease	1.11 (1.03, 1.19)	0.009	1.15 (1.08, 1.23)	<0.001
Moderate or severe liver disease	1.40 (1.14, 1.72)	0.001	1.43 (1.16, 1.74)	<0.001
Chronic pulmonary disease	1.04 (0.94, 1.15)	0.4		
AIDS/HIV	0.88 (0.60, 1.25)	0.5		
Inflammatory bowel disease	1.10 (0.95, 1.27)	0.2		
Charlson Comorbidity Index				
0–1	ref			
2–3	1.07 (0.97, 1.17)	0.2		
≥4	1.05 (0.97, 1.14)	0.2		
ICU admission	0.66 (0.59, 0.75)	<0.001	0.66 (0.59, 0.75)	<0.001
	AUC = 0.56, Accuracy = 0.84		AUC = 0.56, Accuracy = 0.84	

^1^ OR: odds ratio, CI: confidence interval, AUC: area under the curve, ref: reference.

## Data Availability

A contract signed with the Spanish National Health System, which provided the dataset, prohibits the authors from providing their data to any other researcher. Furthermore, the authors must destroy the database upon the conclusion of their investigation. The database cannot be uploaded to any public repository. However, researchers can contact the corresponding author or apply for a data extraction request at: https://www.sanidad.gob.es/en/estadEstudios/estadisticas/estadisticas/estMinisterio/SolicitudCMBD.htm (accessed on 30 November 2024).
